# Human Amniotic Epithelial Stem Cells: A Promising Seed Cell for Clinical Applications

**DOI:** 10.3390/ijms21207730

**Published:** 2020-10-19

**Authors:** Chen Qiu, Zhen Ge, Wenyu Cui, Luyang Yu, Jinying Li

**Affiliations:** 1 MOE Laboratory of Biosystems Homeostasis & Protection and College of Life Sciences-iCell Biotechnology Regenerative Biomedicine Laboratory, College of Life Sciences, Zhejiang University, Hangzhou 310058, China; chenqiu@zju.edu.cn (C.Q.); cui_wenyu@zju.edu.cn (W.C.); 2 Institute of Materia Medica, Hangzhou Medical College, Hangzhou 310013, China; gezhen@zjams.com.cn

**Keywords:** human amniotic epithelial stem cells, cell therapy, plasticity, low immunogenicity, paracrine effect, clinical application, perinatal stem cells

## Abstract

Perinatal stem cells have been regarded as an attractive and available cell source for medical research and clinical trials in recent years. Multiple stem cell types have been identified in the human placenta. Recent advances in knowledge on placental stem cells have revealed that human amniotic epithelial stem cells (hAESCs) have obvious advantages and can be used as a novel potential cell source for cellular therapy and clinical application. hAESCs are known to possess stem-cell-like plasticity, immune-privilege, and paracrine properties. In addition, non-tumorigenicity and a lack of ethical concerns are two major advantages compared with embryonic stem cells (ESCs) and induced pluripotent stem cells (iPSCs). All of the characteristics mentioned above and other additional advantages, including easy accessibility and a non-invasive application procedure, make hAESCs a potential ideal cell type for use in both research and regenerative medicine in the near future. This review article summarizes current knowledge on the characteristics, therapeutic potential, clinical advances and future challenges of hAESCs in detail.

## 1. Introduction

Cell therapy is an efficient way to abate patients’ suffering and to ameliorate a wide range of diseases and injuries to some degree. With a long history of research, cellular therapy is becoming one of the most promising fields in regenerative medicine [1]. There are many potential cell sources for regenerative medicine, such as embryonic stem cells (ESCs) and induced pluripotent stem cells (iPSCs). Inspired and encouraged by the discovery of ESCs [2,3], the methods of differentiating ESCs into the three germ layers have been further developed [4,5,6,7], initiating a wide range of applications from bench to bedside. Twenty-five years after the first discovery of embryonic stem cells, the generation of iPSCs opened the door to a brand-new world of methods of direct differentiation and transplantation therapy, reducing ethical risks. However, the risk of teratoma formation still exists in both ESC and iPSC transplantation, and this is the major concern for further clinical application [8,9]. Of note, the issue of graft rejection using these two kinds of cells should also be taken seriously [10,11]. Although methods of isolation and differentiation of multiple kinds of somatic stem cells have been further established to limit the tumorigenicity of stem cell therapy, ethical concerns and a lack of large-scale cell supply limits their clinical application [12].

The placenta used to be discarded postpartum as medical waste, but it is now regarded as an exploitable source of pluripotent stem cells, according to increasing evidence collected during the last dozen years. Various stem cell types have been isolated from the placenta, such as human amniotic mesenchymal stromal cells (hAMSCs), human umbilical cord mesenchymal stromal cells (hUMSCs), and human amniotic epithelial stem cells (hAESCs) [13,14]. As placenta-derived cells, these stem cells have common advantages, such as an abundant supply, minimal ethical issues, and low DNA damage, which make them an ideal cell source for cell transplantation and regenerative medicine in the clinic.

In this article, we focus on hAESCs, a kind of human placental stem cells derived from fetal membranes. Firstly, we demonstrate the pluripotent epiblast origin of human amnion and hAESCs and introduce existing protocols for isolating and culturing hAESCs. Specifically, we introduce a novel serum-free system for hAESCs isolation and culture, aiming to adhere to clinical standard and to promote the clinical translation of hAESCs. Then, we discuss the characteristics of hAESCs, especially their stem-cell-like plasticity, safety (including non-tumorigenicity and low immunogenicity), and paracrine effect. The unique characteristics of hAESCs, non-tumorigenicity and little ethical concerns, are their major advantages compared with ESCs and iPSCs [15,16] and make them attractive for clinical application. Finally, we summarize the clinical trials reported so far involving hAESCs and discuss future challenges for amnion-membrane-derived stem cell research.

## 2. The Origin of the Human Amnion and Amniotic Epithelial Stem Cells

The placenta, as the first organ involved in fetal development, plays an essential role in gestation and parturition. It connects the developing fetus to the maternal uterine wall via the umbilical cord, allowing gas, fetal waste, nutrients, and other materials necessary for fetal development to be exchanged between the mother and fetus [17]. Another fundamental role of the placenta is to establish a suspended environment for the developing embryo against pressure and distortion from adjacent tissues. Meanwhile, the placenta encases the amniotic fluid and fetus, providing immunoprotection and a suitable site for growth and development. Furthermore, owing to its multilayer structure and elasticity, the placenta has the capacity to secrete hormones that can support gestation and to alleviate stress on the fetus during pregnancy [12,18,19].

The placenta is composed of three layers: the amnion, chorion, and decidua. The fetal part of the placenta consists of the amnion and chorion, which are derived from the inner cell mass of blastocysts and trophoblasts respectively. On the other hand, the decidua, a structure full of maternal cells with an abundant extracellular matrix, forms the maternal placenta [20,21]. The fetal membranes facilitate the exchange of gas, nutrients, and waste, serving as a barrier to protect fetus from the maternal immune system and synthesizing certain hormones and enzymes that are critical during pregnancy and parturition [19,21,22,23].

From the perspective of development, the origins of hAESCs and other components of the placenta are fundamentally different. Human amnioblast, the precursor of hAESCs, is derived from the pluripotent epiblast at around 8 days after fertilization, whereas other fetal parts of the placenta are derived from the trophectoderm. This might reflect the uniqueness of hAESCs compared with other types of perinatal pluripotent cells. According to a classic anatomic study by Bourne [24], the amnion is a thin, avascular membrane that consists of five layers: the epithelium layer, the basement membrane layer, the compact layer, the fibroblast layer, and the spongy layer. The epithelium layer is the innermost single-cell layer that contacts the amniotic fluid directly. Although these layers seem to serve as a whole during pregnancy, they are quite different from development aspects. After the partial implantation of blastocysts into the endometrial stroma, the inner cell mass splits and forms the embryonic disc, which is composed of the epiblast and hypoblast [25]. The epiblast, the source of all three germ layers, differentiates into the developing embryo eventually [26]. Meanwhile, a cavity, called the amniotic cavity, appears between the epiblast and cytotrophoblast. It is assumed that the primordial cells, lining the amniotic cavity and termed amnioblasts, are the result of epiblast cell division. The processes of cell division and amnioblast formation occur prior to gastrulation, the period of cell fate determination, which means that the amnioblasts do not belong to any germ layers and theoretically seem to be pluripotent. After the following development and cell differentiation, the amnioblasts eventually differentiate into amniotic epithelial stem cells (AESCs). Thus, it is reasonable to speculate that the AESCs maintain pluripotency until term.

## 3. The Establishment of Serum-Free Culture System for Human Amniotic Epithelial Stem Cells

The isolation and culture protocols of hAESCs from term placenta have been developed over a long period of time, starting with the pioneering work by C.A. Akle et al. [27]. Based on Akle’s work, Miki et al. modified the protocol, establishing a three-step digestion method for the isolation of hAESCs and cultured hAESCs in standard culture media supplemented with 10% fetal bovine serum, L-glutamine, nonessential amino acids, 2-mercaptoethanol, sodium pyruvate, and antibiotic-antimycotic. In the presence of epidermal growth factor (EGF), cultured hAESCs display a typical cobblestone-shaped epithelial phenotype [16]. Of note, nearly 200 million hAESCs can be harvested from one human term amniotic membrane, allowing sufficient cell supply for clinical use [16]. More importantly, for clinical purposes, it is fundamental to adopt the current good manufacturing practices (cGMP) to meet clinical demand, as mentioned by Gramignoli in 2016 [28]. A detailed and normative protocol is described in the paper, followed by quality assessment criteria to guarantee that cell products can be used for both clinical and basic research.

As mentioned by previous studies, hAESCs are generally cultured with fetal bovine serum to ensure rapid growth and proliferation. However, serious concern has been raised about the utilization of animal serum in clinical applications. To establish a more effective protocol for hAESC culture in accordance with current guidelines for clinical use, our lab developed a serum-free system for hAESC isolation and culture [29]. In brief, hAESCs are cultured with complete culture medium containing F12/DMEM, 10% KSR (KnockOut Serum Replacement), 2 mmol/L L-glutamine, 1% nonessential amino acids, 55 μmol/L 2-mer-captoethanol, 1 mmol/L sodium pyruvate, 1% antibiotic–antimycotic, and 10 ng/mL EGF. Notably, the hAESCs cultured with the serum-free system show a steep growth curve after passage 1 and a higher level of proliferation ability around passages 2 and 3 compared with traditional serum culture. Besides, the pluripotency and low immunogenicity are maintained in the serum-free condition tested by the expression of NANOG, OCT4, and low levels of HLA-DR and HLA-DQ. Additionally, Yang and colleagues showed that hAESCs sustain potential immunoregulatory ability in serum-free culture media and determined that CD90-positive hAESCs, a special population in the primary cells, are the major contributor to TNF-α inhibition and IL-10 promotion compared with other hAESCs. Moreover, a safety evaluation of the serum-free system at the preclinical level has been implemented. Results showed that little toxic response is induced by hAESC injection, and no hemolysis and allergic symptoms are observed in animal models. In addition, tumorigenicity is another critical criterion for clinical application, and results indicated that no tumor occurrence is detected in the immunodeficient mice (NOD/SCID) after hypodermic injection of hAESCs, presumably due to the lack of telomerase hTERT in hAESCs.

In conclusion, hAESCs show better growth behavior and regular morphology in the serum-free system even after undergoing cryopreservation. Besides, hAESCs still maintain their properties such as pluripotency, weak immunogenicity, and modest immunoregulatory in the serum-free condition. Meanwhile, hAESCs show a negative result for tumorigenicity, meeting the critical requirement of the safety evaluation for the clinical use of hAESCs. All these results indicated that the developed integrated system could prevent serum-derived contamination by excluding animal source constitutions, which aims to facilitate further clinical translation of hAESCs.

Besides human AESCs, there are mounting studies on AESCs derived from other species such as rat and sheep, which could potentially provide a route to investigate the characteristics of AESCs at different gestational stages and the difference between different species. Table 1 summarizes some key points of methods for isolating AESCs from different species. The results of these studies indicate that, though derived from different species, AESCs still possess basic properties similar to human AESCs such as pluripotency and low immunogenicity. Hence, such methods can be of real significance in evaluating the therapeutic effect of AESCs in allotransplantation, which contributes to avoid the rejection associated with mismatch.

## 4. The Plasticity of Human Amniotic Epithelial Stem Cells as Clinical Tool Cells

In contrast to other parts of the placenta, human amniotic epithelial stem cells are derived from the pluripotent epiblast at around 8 days after fertilization, which happens prior to gastrulation; thus, it is reasonable to hypothesize that a certain proportion of hAESCs may retain the plasticity of pregastrulation embryo cells. Indeed, the latest research revealed that the pluripotent factors NANOG, OCT4, and SOX2 are not fully silenced in hAESCs, as their promoters are only partially methylated, displaying a similar epigenetic profile compared with hiPSCs, and their downregulation is regulated at the post-transcriptional levels by specific miRNAs [36]. Meanwhile, Miki et al. reported that most hAESCs express some ESC-associated pluripotent markers, such as OCT-4, NANOG, SSEA-3, SSEA-4, TRA-1-60, and TRA-1-80, in the early stage [16]. Although these stem cell markers are lost over time, some AE cells may retain these stem cell markers at term.

hAESCs represent a heterogeneous cell population according to profiling of different stem cell markers [26]. For example, the most stringent stem cell marker transcription factor, NANOG, is expressed on only 1–3% of hAESCs, while about 50% of hAESCs remain SSEA-4-positive at term [16]. Co-expression of stem cell surface markers SSEA-4, TRA1-60, and TRA1-81 is found on only about 4% of AE cells [37]. Some studies have confirmed that stem cell marker positive hAESCs are scattered randomly over the whole amnion membrane and appear to be surrounded by marker-negative cells, rather than existing in a cluster manner [26,38,39]. Moreover, another recent study demonstrated that hAESCs from different areas of amnion membrane (AM) have differences in pluripotency marker expression and proliferative ability [40]. These data demonstrate that the whole amniotic epithelium is covered with hAESCs at different development stages with diverse gene expression profiles and various degrees of stemness [37]. However, it is reported that most primary hAESCs, even those that are stem cell marker negative, can be induced towards specific differentiation in vitro [41]. With heterogeneity, though, hAESCs have the potential to differentiate into all three germ layers under proper conditions [15,16].

In previous studies, the formation of some small cell clusters or spheroids, which are similar to the embryoid bodies derived from ES cells, were found after an adjacent monolayer of hAESCs is cultured in vitro for several weeks. Intriguingly, stem cell markers could be detected for a long term in the spheroid structures, and cells in the core of the cluster are more likely to be pluripotent-marker-positive, suggesting that the spheroid structures could better support and retain the stemness of hAESCs in vitro [26].

The most convincing evidence to support the pluripotency of amniotic epithelium (AE)-derived stem cells is the generation of chimeric animals. Tamagawa et al. injected AE-derived cells into an early-developing mouse blastocyst and generated an aggregation chimera, in which the AE-derived cells were distributed among all three germ layers [42]. In all, the pluripotency of hAESCs has been confirmed using both in vivo and in vitro models.

### 4.1. Differentiation into the Ectoderm

Amniotic tissues and hAESCs have been utilized to treat neurodegenerative diseases since the 1990s owing to their pluripotency and secretion of neurotrophic factors, which are the two expected mechanisms of cell transplantation. It has been revealed that more than 50% of hAESCs are positive neural cell markers, with 20–30% of cells having positive glial cell markers [16,43,44]. Sakuragawa et al. systematically summarized the neural cell markers expressed by hAESCs, such as MAP2 and GFAP, and suggested that all-trans retinoic acid (RA) and fibroblast growth factor-4 (FGF-4) could induce the expression of some specific neural-specific genes [43]. Furthermore, it has been reported that noggin, bFGF, and RA could promote the transformation of hAESCs into neural cells, but serum could reduce the ratio [45].

In addition to the investigation of neural markers in vitro, implantation of hAESCs has been carried out on the animal models. The capability of AESCs to differentiate into neuron-like cells was confirmed after transplantation of neural stem cell marker positive rat AESCs into the hippocampus of gerbils for 5 weeks [46]. Another study demonstrated that amnion-derived stem cells injected into the developing fetal rat brain in utero could migrate throughout the majority of brain, displaying some neuronal morphologies on embryonic day 21.5 [47]. In addition, under pathological settings (brain edema, Parkinson’s disease, traumatic brain and spinal cord injury, etc.), implanted hAESCs have also been confirmed to be able to differentiate into MAP2-positive neuron-like cells [48,49,50,51]. 

In addition to the potential of differentiation, it is worth noting that hAESCs are able to synthesize and secrete some neurotrophic factors. A body of evidence indicates that hAESCs are capable of secreting acetylcholine (Ach) [52], catecholamine (CA) [53], dopamine (DA) [54], brain-derived neurotrophic factor (BDNF), neurotrophin-3 (NT-3) [55], activin, and noggin [56]. Moreover, it has been indicated that hAESCs could produce certain neurotrophic factors that have a neuroprotective effect on rat retinal ganglion cells and neural stem cells when co-cultured with them [57,58]. Later, Meng et al. [59] and Sankar et al. [60] provided evidence that hAESCs are able to promote the survival of neurons and remodel the microenvironment for neural repair. 

Owing to these factors secreted by hAESCs, which are essential to the fundamental functioning of the brain, more and more studies have focused on the treatment of Parkinson’s disease (PD) with hAESCs. PD is a neurodegenerative disease, which is characterized by the progressive loss of midbrain substantia nigra dopaminergic neurons and affects both peripheral and central nervous systems [61]. Consequently, there is accumulating evidence regarding the curative effect of hAESC transplantation into PD animal models [62,63,64]. Yang et al. [64] stated that hAESCs survive for 10 weeks and a portion of them differentiate into tyrosine hydroxylase (TH)-positive neurons, with significant alleviation of PD symptoms and reduction of the loss of neurons in the substantia nigra.

### 4.2. Differentiation into the Mesoderm

Due to the insufficient regeneration of adult cardiomyocytes, it is important to find a suitable cell source for cellular therapy for severe heart diseases such as progressive heart failure. hAESCs may also be capable of differentiating into cardiomyocytes. Miki et al. [16] indicated that hAESCs express cardiac-specific genes and transcription factors such as MLC-2, GATA-4, and Nkx 2.5 over 14 days in culture medium supplemented with 1 mM ascorbic acid. Amnion-derived cells were transplanted into the left ventricular myocardium of post-infarcted rats, and the results showed that the injected cells expressed some cardiac markers and appeared to partially differentiate into vascular smooth muscle cells and cardiomyocytes [65]. Furthermore, Ilancheran et al. proved that some cardiomyocytic markers—GATA-4, ANP, MYL7, CACNA1 C, KCND3, and TTNT—could be detected after hAESCs were cultured in an induced medium, and an ultrastructural analysis of the cultured cells showed similar results to mature cardiomyocytes [15]. Ilancheran also mentioned that hAESCs can be induced into other mesodermal cell lineages, such as adipocytes and osteocytes, under proper conditions.

### 4.3. Differentiation into the Endoderm

Studies of hAESC differentiation into endoderm cells have generally focused on hepatic and pancreatic cell lineages. The insufficient supply limits cell replacement therapies being used in clinical applications. As for the generation of functional pancreatic cells, the differentiated hAESCs have been proven to express specific markers that are necessary for the development and function of pancreatic islets, and they have been shown to secrete classical endocrine hormones such as insulin and glucagon [66,67,68,69]. In vivo studies have confirmed that hAESC-induced pancreatic cells have normal function after being transplanted into diabetic model mice. Intriguingly, these mice showed normal blood glucose levels and body weights one month after implantation. These data indicate that hAESCs are capable of differentiating into functional insulin-producing cells, and they normalize the physiological index of diabetic mice, eventually prolonging their life span. Notably, one recent study reported that hAESCs form insulin-producing 3D spheroids with a high cytoplasm complexity and intense protein secretion after 20 days of pancreatic induction in serum-free media [70], broadening the application of hAESCs as micro-organs with the assistance of biomaterials. 

In the field of hepatocyte regeneration, in spite of the pioneering work utilizing ES cells and bone marrow cells [71,72], their applications are still limited due to the incidence of teratomas and inconvenient accessibility. There is increasing evidence showing that hAESCs could successfully differentiate into functional hepatocytes and are safer and more feasible for use clinically. In a previous study, Miki et al. proved that steroid hormones are able to induce hepatic differentiation of hAESCs and that dexamethasone is the most efficient agent for increasing the expression of hepatic-specific markers [73]. Recently, Quan-Wen Liu et al. successfully generated hepatocyte-like cells (HLC) from hAESCs using a three-step, 14-day protocol, and the liver function and survival rate of CCl_4_-induced acute liver failure (ALF) mice was significantly improved after HLC transplantation [74]. Meanwhile, it has been stated that hAESCs tend to engraft in the host liver and differentiate into hepatocyte-like cells after transplantation [75,76], and the expression level of hepatocyte-specific genes is comparable to those in the fetal liver [77].

In addition, research has shown that hAESCs could differentiate into cholangiocytes [78] and lung epithelial cells [41], indicating that the transplantation of hAESCs might alleviate and provide potential therapeutic options for cholestatic ductal hyperplasia and lung fibrosis, respectively. 

Taken together, hAESCs express various lineage-related markers, which represent their potential to differentiate to several cell lineages as progenitor cells. The differentiation potential of hAESCs into cell types derived from all three germ layers has been widely reported, and we briefly summarize these studies in Table 2.

However, the expression percentage of surface and molecular pluripotency markers remains variable due to the passage numbers and measuring methods. The fresh isolated hAESCs have a low proliferation efficiency and stop growing after 4–5 passages due to DNA damage or shortened telomeres, which impair their clinical application in regenerative medicine [79]. To meet clinical needs, Kaixuan Zhou et al. established immortalized human amniotic epithelial stem cells (iHAESCs) via introducing viral oncogenes E6/E7 and human telomerase reverse transcriptase (hTERT). They found the immortalized HAESC cells with an extended life span retained characteristics of the original cells, including pluripotent markers and differentiation capabilities [79]. Recently, they showed that the immortalized HAESC cells have relatively higher resistance to oxidative damage compared with immortalized human amniotic mesenchymal cells (iHAMs) because of higher level of basal antioxidant factors (SOD2, Nrf2, HO-1), suggesting AESCs provide protection against oxidative stimulation under oxidative stress [80]. These results offer more choices for their cell therapy and regenerative medicine, reflecting the plasticity of AESCs as a potential clinical tool cell source.

## 5. The Safety Evaluation of Human Amniotic Epithelial Stem Cells

As mentioned above, the plasticity confers the possibility of clinical applications to hAESCs by producing several functional cell types. In clinical trials, however, safety is the bottom line for everything. Based on the premise, the safety evaluation of hAESCs has attracted increasing attention. In this part, we focus on the characteristics of both toxicity and tumorigenicity as well as weak immunogenicity.

### 5.1. Toxicity and Tumorigenicity

Besides the consideration of animal serum-derived contamination, our previous work also confirmed that neither acute nor long-term toxicity response is triggered by hAESC injection in xenogeneic transplantation [29]. Specifically, little change in T cell population and corelated cytokines is observed after high-dose (10^8^ cells/kg) hAESC injection into mice, indicating that hAESCs do not disturb the immune system. Moreover, Yang et al. also demonstrated hAESCs have no adverse effect on body weight, viscera weight, blood parameters, and coagulation indexes. Hence, no toxicity caused by hAESCs was observed in our systematic safety evaluation.

Tumorigenicity, another major concern for stem cells, limits a large proportion of translation from bench to bedside. Given that mesenchymal stem cells were reported to promote tumor growth [93], another critical part of safety evaluation is to identify whether hAESCs have an effect on the tumor generation and promotion. In previous studies, the non-tumorigenicity of AESCs has been confirmed in many species, including in humans [27,75,76]. Based on these results, we further injected hAESCs into immunodeficient mice subcutaneously with both low (2 × 10^6^ cells) and high (4 × 10^6^ cells) doses, and results showed that no tumor occurrence is observed after 4 weeks post-injection [29]. Mechanistically, the expression of telomerase hTERT in hAESCs is not detected, explaining why hAESCs do not generate tumors. Notably, several studies demonstrated that hAESCs could inhibit tumor cell growth and induce apoptosis of cancer cells, including HeLa cells and breast and ovarian cancer cells [94,95,96]. However, Yang et al. reported that co-injection of hAESCs and Raji tumor cells into immunodeficient mice shows a similar survival duration when compared with the mice receiving only Raji cells, which indicated hAESCs have little effect on tumor growth [29]. Therefore, whether hAESCs possess a potential anticancer property, and the molecular mechanism of this process, remains to be further determined.

### 5.2. Low Immunogenicity

According to the hypothesis of Medawar (1953) about the mechanism of unresponsiveness that exists between the fetus and maternal immune system [97], the placenta can be regarded as a physical barrier that isolates the fetus from maternal immunity. Indeed, one of the roles of the placenta is to protect the fetus from being attacked by the maternal immune system during pregnancy. However, the placenta does not work as a mere physical barrier without any connection between fetal tissue and the maternal system. The chorion villi were found to be immersed in the maternal blood, and fetal cells were detected in the maternal circle long after pregnancy completion [98,99], indicating that despite direct contact between fetus cells and the mother, immune-unresponsiveness is the result of the characteristics of certain fetal cell types [100]. 

The immune privilege of amnion and amnion-derived cells has been confirmed by direct implantation into volunteers and animals without acute rejection in vivo. Monolayer amniotic membrane and hAESCs have been implanted into an immunotype-mismatched human subcutaneous pocket [27], rat limbal area, intracorneal space, kidney capsule [101], guinea pig cochlea [102], monkey spinal cord [60], and mice anterior chamber [103]. All of these studies showed that amniotic tissue and hAESCs are able to survive in the host for a long time without displaying any infiltration or rejection of host immune cells, suggesting that amnion-derived cells, including hAESCs, have low immunogenicity. 

Mechanistically, due to the low immunogenicity of hAESCs reported, it is hypothesized that hAESCs have a unique expression profile of HLA antigens, which are a series of polymorphic antigens expressed on the cell surface and coded by a complicated gene system in humans. It was initially reported that hAESCs did not express HLA-I (HLA-A, -B, and -C and β2 microglobulin) and HLA-II molecules [27]. However, with advanced techniques, it was later confirmed that hAESCs do express lower levels of HLA-I antigens than those found on other placental cells and somatic cells [104,105]. Further identifying the HLA antigen profile of hAESCs, a body of evidence indicated that all three classical HLA-I molecules (-A, -B, and -C) can be identified on the hAESCs [106,107], even after cryopreservation [108] or passages 4–5 (P4-P5) [109]. Recently, Strom et al. summarized the expression profile of HLA molecules on hAESCs, including the expression of both classical HLA-I (-A, -B, and -C) and the relatively lower-expression of non-classical HLA-I molecules (-E, -F, and -G) [110]. In addition, no HLA-II molecules (-DR, -DQ, and -DP) were detected on hAESCs. Among these HLA antigens, classical HLA-I and all HLA-II are regarded as transplantation-related antigens because of their high polymorphism, which contributes to immune recognition and rejection during allograft. On the other hand, non-classical HLA-I molecules have the capacity to inhibit the immune reaction under both autoimmune and transplantation conditions, owing to their different polymorphism, expression, structure, and function compared with classical HLA-I and HLA-II antigens [111,112]. Consequently, this special expression profile of HLA molecules has been suggested as the mechanism of the low immunogenicity and seems to explain why hAESCs do not trigger a severe immune reaction after allogeneic or even xenogeneic transplantation. 

Interestingly, among those non-classical HLA-I antigens, HLA-G, which was first discovered in the placenta tissue [113], has received much more attention since Hammer’s report claiming that it may play a role in suppressing the maternal immune response [106]. Indeed, hAESCs express HLA-G, and its expression significantly increases after amniotic cell treatment with IFN-γ (interferon-gamma) [114]. Furthermore, a study reported that term hAESCs express HLA-G at twice the level of preterm cells [115]. For hAESCs, HLA-G molecules are expressed in both membrane-bound forms (HLA-G1 to -G4) and soluble forms (HLA-G5 and HLA-G6) [101], which may explain why HLA-G molecules can be detected in amniotic fluid [106]. It has been reported that HLA-G is able to inhibit the proliferation of lymphocytes in a mixed lymphocyte reaction [116]. In graft-versus-host disease (GVHD), Le Maux et al. found that the high expression of soluble HLA-G is related to a certain regulatory T (Treg) cell content in the host blood, which is usually regarded as being associated with a good prognosis in allogeneic transplantation [117]. It has been hypothesized that soluble HLA-G may create an immune-tolerogenic environment that improves allograft survival after transplantation. Furthermore, it has been shown that only two receptors on natural killer (NK) cells are involved in HLA-G recognition, which might induce sufficient but not excessive inhibition of the immune response to maintain the process of pregnancy [118].

In summary, studies have revealed the absence of HLA-II antigens and co-stimulatory factors on hAESCs, yet there is still controversy over whether hAESCs express HLA-I molecules. The expression of HLA-G on hAESCs was considered to play a vital role in suppressing the maternal immune response. More details about the special expression of non-classical HLA-I antigens on hAESCs and the relationship between classical HLA-I and the immune response should be investigated further.

## 6. The Paracrine Effect of Human Amniotic Epithelial Stem Cells

The immunoregulatory characteristics of hAESCs and human amnion tissue, which are mainly attributable to their paracrine effect, have been reported for a long time. These include antibacterial, proinflammatory, and anti-inflammatory properties, which play pivotal roles in different situations. The properties of anti-inflammation and proinflammation, which seem contradictory to each other, have already been put forward for the behavior of mesenchymal stromal cells in a process called “licensing”, which means these cells are able to play different roles depending on the inflammatory milieu [119]. 

### 6.1. Antibacterial and Proinflammatory Properties

During pregnancy, the placenta and amniotic fluid provide an ideal environment and serve as a cushion for fetal growth. However, preterm birth can be a serious accident with multiple causes. Among these causes, intra-amnion infection is the major cause of spontaneous neonatal preterm birth (PTB) and preterm premature rupture of the fetal membranes (PPROM) [120], which impairs pulmonary and neurodevelopmental function [121]. According to the natural mechanism of maternal defense against infection, it is reasonable to hypothesize that amnion tissue and hAESCs may obtain certain antibacterial and proinflammatory capabilities during pregnancy. The antimicrobial function of amniotic fluid was first reported in 1970 with the discovery of pathogenic inhibitors, including lysozyme, transferrin, immunoglobulin, and β-globulin, in the liquid environment [122,123]. Furthermore, as the first line of defense against intra-amnion infection, amniotic epithelial cells have been confirmed to express β3-defensin, which is specifically upregulated with the stimulation of lipopolysaccharide (LPS) [124]. Fas, on the other hand, which is a member of the nerve growth factor receptor–tumor necrosis factor receptor family, serves as an apoptosis-signaling molecule that exists in both membrane-bound and soluble forms [125]. The Fas antigens expressed on the cell surface are able to trigger apoptosis of the target cells bound with Fas ligand or anti-Fas antibody. However, soluble Fas, an alternatively spliced product of the Fas gene, protects the target cells from Fas-mediated apoptosis by neutralizing binding between membrane-bound Fas and the Fas ligand [126]. It has been proven that the concentration of soluble Fas in the amniotic fluid is high [127]. In addition, Fas has been shown to be expressed predominantly in epithelial cells throughout the whole amnion, so it is reasonable to hypothesize that hAESCs are one of the major sources of soluble Fas existing in the amniotic fluid [128]. Due to its anti-apoptosis function, it is believed that the soluble Fas in amniotic fluid plays a pivotal role in maintaining the activity of leukomonocytes and inhibiting apoptosis to provide protection from infection [129]. Interestingly, it has been verified that hAESCs express distinct functional Toll-like receptors 5 (TLR5), TLR6/2, and TLR4, which are crucial regulators of the innate immune system [120]. As a result of the activation and recognition of pathogen-associated molecular patterns (PAMPs) by TLRs (TLR5, TLR6/2), hAESCs are able to secrete several proinflammatory cytokines such as IL-6 and IL-8 to balance homeostasis for fetal growth. The hAESC barrier is able to recognize and respond to pathogens through the function of TLRs, which makes sense in vivo.

### 6.2. Anti-Inflammation

Although the antibacterial and proinflammatory characteristics are essential to protect the fetus from infection during pregnancy, the major function of hAESCs, which is the most commonly utilized in preclinical and clinical trials, is anti-inflammation. It was reported that hAESCs and amnion tissue have amazing anti-inflammation characteristics with a long history of application. In brief, hAESCs and amnion-derived cells may suppress lymphocyte responsiveness without inducing mixed lymphocyte reaction via the secretion of TGF-β and prostaglandin E2 (PGE2) [129,130]. Moreover, hAESCs exert multiple immunosuppressive activities via inhibiting the cytotoxicity of NK cells and monocytes and promoting the induction and maturation of Treg cells [131,132]. Recently, researchers found that hAESCs can improve the phagocytic ability of neutrophils while reducing their oxidative burst capacity, thereby protecting the fetus from both microbial infections and oxidative stress and the consequent inflammation [133].

It is rather complicated to illustrate the deep mechanisms by which hAESCs suppress an excessive immune response. It was reported that hAESCs are capable of preventing macrophage and T cell infiltration into the lesion area and maintaining the proper portions of different T cell subtypes by modulating the cytokine concentration inside the microenvironment in certain autoimmune disorder models [134,135]. Actually, previous research has focused on the anti-inflammation effects of conditioned medium and cytokines from hAESCs. Li et al. found that the supernatant of hAESC culture medium could significantly suppress the proliferation and activity of neutrophils and T and B lymphocytes [136]. Meanwhile, it can also induce the apoptosis of lymphocytes and the polarization of macrophages from M1 to M2. The latter is considered to be a pivotal subtype in inhibiting inflammation and encouraging tissue repair. Three types of cytokines related to anti-inflammation have been proven to be produced by hAESCs and amniotic tissue: (1) apoptosis inducers such as tumor necrosis factor-α (TNF-α), Fas ligand (FasL), and tumor necrosis factor-related apoptosis-inducing ligand (TRAIL); (2) macrophage migration inhibitors such as transforming growth factor-β (TGF-β) and macrophage migration-inhibitory factor (MIF); and (3) other antiangiogenic and anti-inflammatory proteins including interleukin-1 receptor antagonist (IL-1ra), interleukin-10 (IL-10), tissue inhibitors of metalloproteinase (TIMPs), collagen XVⅢ, and thrombopondin-1 [134,135,136,137,138]. Data indicate that these cytokines are critical in the hAESC- and amniotic-tissue-mediated anti-inflammation process. However, Wolbank et al. proved that direct cell-to-cell contact is also an essential prerequisite for hAESC-mediated immunosuppression within the trans-well system, which seems contrary to the function of the cytokines [108]. Under physiological and pathological conditions, the microenvironment may be rather intricate, involving both cytokine secretion and direct cell-to-cell contact, in order to maintain proper immune status for fetal development. Apart from cytokines, some complementary inhibitory membrane proteins that have been detected on hAESCs may be another mechanism of immunosuppression. Rooney et al. reported that hAESCs express all three types of complementary inhibitory membrane proteins, including CD59 antigen, decay-accelerating factor (DAF), and membrane attack complex inhibitory protein (MIP) [139]. The functions of these three proteins have been identified in previous studies that indicated their profound importance in protecting cells from complement-mediated lysis [140,141,142]. Consequently, the significance of these three proteins expressed on hAESCs is self-evident, and these proteins might contribute to the anti-inflammation role of hAESCs in the allotransplantation and wound healing process.

Above all, hAESCs might play totally different roles according to the microenvironment. During pregnancy, without a high level of inflammatory signals, hAESCs may adopt a proinflammatory phenotype that could serve as a part of the innate immune response to protect the fetus from the pathogen invasion via secreting certain factors. However, in a highly inflammatory environment, the anti-inflammation property would be considered to play the predominant role of hAESCs though the secretion of soluble factors. When hAESCs are used as grafts in regenerative medicine, the anti-inflammation property could contribute to the survival of donor cells and the healing of wounds. Consequently, the seemingly contradictory properties of hAESCs are dependent on different situations and could be utilized accordingly. 

Notably, recent studies have focused on amnion epithelial stem cell derived exosomes as a new paracrine mechanism and found that exosomes derived from hAESCs play a vital role in the pathogenesis of human parturition and preclinical treatment. Samantha Sheller et al. described the classic shape, size, and markers (CD9, 63, 81, HSC 70, Nanog) of the hAESC exosome and found that increased colocalization of H3, HSP70, and active p38 (P-p38) MAPK in AESC exosomes reflect the physiologic status of the cell in response to oxidative stress [143]. Then, they further found primary AESC exosomes packaging high-mobility group box 1 (HMGB1) and cell-free fetal telomere fragments (cffTF) translocated form nuclei to cytoplasm under oxidative stress (OS) conditions, which may act as a fetal signal at term [144]. Recently, Emily E. Hadley et al. demonstrated that hAESC-released exosomes under control and oxidative stress conditions were taken up by myometrial, decidual, and placental cells. The exosomes from both conditions significantly increased inflammatory mediators (IL-6, IL-8, and PGE2) and activated NF-kB in maternal cells, indicating that hAESC exosomes are a novel paracrine mechanism of fetal–maternal communication [145]. In terms of preclinical treatment with hAESC exosomes, researchers presented strong evidence on wound healing and fibrosis. Bin Zhao et al. found hAESC-exosome-derived miRNAs promoted the proliferation and migration of fibroblasts and further accelerated wound healing [146,147]. Moreover, a recent study demonstrated that hAESC exosomes accelerated diabetic wound healing by promoting angiogenesis and fibroblast function via activation of the PI3K–AKT–mTOR pathway [148]. Moreover, Jean L. Tan et al. found that hAESC exosomes reduced liver fibrosis and bleomycin-induced lung injury and enhanced endogenous lung repair by polarizing and increasing macrophage phagocytosis, reducing neutrophil myeloperoxidases, and suppressing T cell proliferation directly [149,150].

## 7. The Therapeutic Potential and Clinical Advances of Human Amniotic Epithelial Stem Cells

Based on their plasticity and immunomodulatory properties, hAESCs have been considered for treatment of a broad variety of conditions, including lung fibrosis [40,115,131,149,151,152,153,154], spinal cord injuries [60], Parkinson’s disease [64], Alzheimer’s disease [155], diabetes [69], wound healing [147,148,156], liver fibrosis [75,157,158,159], autoimmune uveitis [135], Hashimoto’s thyroiditis and systemic lupus erythematosus [134], and intrauterine adhesions [160,161,162]. Among these preclinical trials involving hAESCs, autoimmune diseases make up a large proportion, presumably because of the tremendous effect of immunomodulation. Indeed, some previous studies by our lab have indicated that hAESCs have obvious curative effect on experimental animal models of autoimmune diseases. In autoimmune uveitis models, hAESCs inhibit the infiltration of macrophages and modulate the balance of T-cell subtypes, leading to the improvement of both local and systemic cytokine environments. To be specific, treatment with hAESCs suppresses the level of IL-17, IFN-γ, and monocyte chemoattractant protein (MCP)-1; meanwhile, the expression of IL-10 is upregulated locally and systemically [135]. Additionally, Tan et al. demonstrated the therapeutic efficacy of hAESCs both in localized and systemic autoimmune disease models. Similarly, hAESCs modulate the balance of TH17 and Treg cells in experimental autoimmune thyroiditis (EAT) and systemic lupus erythematosus (SLE) mice. In addition, hAESCs improved cytokine environment by inhibiting IL-17 A and IFN-γ and enhancing TGF-β [134]. In a word, hAESCs represent a great potential therapeutic strategy against immune diseases in light of their immunoregulatory ability.

Recently, Canciello et al. found that progesterone could prevent the epithelial–mesenchymal transition of amniotic epithelial stem cells and enhance their immunomodulatory properties [163]. Moreover, Hou et al. demonstrated that Vitamin C promotes the proliferation, migration, self-renewal, and paracrine functions and improves the therapeutic potential of hAESCs in premature ovarian insufficiency disease [164]. These promising results indicate that the therapeutic efficacy of hAESCs can be significantly improved by in vitro factor treatment. As described above, these preclinical studies successfully demonstrated the great therapeutic potential of hAESCs as a candidate cell type for regenerative medicine.

In terms of clinical applications, in 1910, Davis was the first to advocate the use of the amniotic membrane as a therapeutic material in skin transplantation [165]. Later, the amniotic membrane was used to treat skin wounds and ulcers, reconstruct the vagina, and prevent tissue adhesion [166,167,168,169,170]. Since the 1960s, the amniotic membrane has gradually been utilized clinically in the field of ophthalmology as an alternative to corneal graft [171,172], where the basement membrane serves as a biological scaffold for epithelial cell migration and the amniotic membrane plays an important role in anti-inflammation. Recently, some clinical trials (ACTRN12618001631291 and ACTRN12618001632280) have been designed to compare the therapeutic effect of amnion with traditional drugs or materials in burn and skin regeneration. Moreover, another trial (ACTRN12619001050145) submitted last year seeks to determine whether the amniotic cells are capable of curing burn wounds by contrasting cellular with acellular amnion. Aside from the fresh amnion tissue, amnion derivates such as amniotic membrane powder have been proposed for wound healing, aiming to develop a rapid and convenient treatment (NCT03754218). Although these clinical trials are just the beginning, the outcome and effect are highly anticipated as an alternative curative method in the future.

Human amniotic membranes and isolated hAESCs have been transplanted into both volunteers [27] and patients with lysosomal storage disease (LSD) since the 1980s, and there have been no reported adverse effects, including tumor formation [173,174,175,176,177]. Tylki-Szymanska et al. reported that after the implantation of the amniotic epithelium in six patients with LSD, three cases showed a diminishment of corneal clouding, and one case displayed a slight increase in plasma beta-galactosidase activity [178]. Scaggiante et al. [175,179] and Bembi et al. [176] injected hAESCs into patients with Niemann–Pick disease (NPD) and found that sphingomyelinase activity significantly increased the symptoms of hepatosplenomegaly and alleviated nutritional malabsorption. No cases of graft-versus-host disease were reported during the follow-up period.

As of September 2020, there were 17 clinical trials listed in the clinical trial database (ClinicalTrials.gov and anzctr.org.au) involving the use of “amniotic epithelial cells” as a biological intervention (Table 3). NCT00344708 describes three cases of persistent corneal epithelial defect (PED) before withdrawal (for potential subjects that did not meet eligibility criteria). In the 6.3-month follow-up period, three patients were observed to show an improvement in clinical behaviors, which was maintained until PED finally vanished, and no vision-loss events occurred [180].

A research group from Monash University, Australia, carried out the first phase I clinical trial to treat bronchopulmonary dysplasia (BPD) with hAESCs (ACTRN12614000174684) and reported initially in 2018 [181] that among six premature infants with bronchopulmonary dysplasia who were given hAESCs intravenously, no adverse effects were observed during a 2-year follow-up period, except for one patient who suffered from transient cardiopulmonary instability during administration. The study suggested that allogeneic hAESCs administration is safe, tolerable, and could improve infants’ respiratory function. A wider-range multicenter dose escalation clinical trial (ACTRN12618000920291) has already been established by this group to confirm the safety and efficacy of intravenous hAESC injection to treat bronchopulmonary dysplasia in 24 subjects.

Notably, hAESCs are rapidly being proposed for use in clinical trials related to the global coronavirus disease 2019 (COVID-19) pandemic onset. Researchers from Monash Health have scheduled a Phase 1 trial (ACTRN12620000676910 p) to assess the efficacy of using hAESCs to treat COVID-19 related respiratory failure and multiorgan complications. They proposed a treatment with 8 million hAESCs per kilogram delivered intravenously according to its registration on Australian New Zealand Clinical Trails Registry (ANZCTR). We anticipate that the clinical trial may be beneficial for the improvement of patients’ conditions.

There are also many more clinical trials utilizing the amniotic membrane, amniotic fluid, amnion-derived cellular cytokine solution (ACCS), and amniotic membrane extract eye drop (AMEED) [182] as biological interventions for therapeutics. However, compared with mesenchymal stem cells (MSCs), which have served as therapeutic cells in hundreds of clinical trials, including some phase III trials, the potential of hAESCs in clinical application has not been fully demonstrated.

## 8. Conclusions and Future Challenges

The placenta is a temporary organ that forms during pregnancy and plays a pivotal role in exchanging gas, nutrients, and waste between the fetus and maternal blood, establishing a suspended and immune-privileged environment for fetal growth and secreting certain hormones that are essential for gestation. Considering the fetal portion in the placenta, it is reasonable to investigate the characteristics of these fetal cells for their potential application value in stem cell therapy and regenerative medicine. HAESCs, derived from the pluripotent epiblast, have advantages over other pluripotent cells in terms of therapeutic benefits. As summarized above, studies have confirmed the plasticity, immunoregulation, and low immunogenicity of hAESCs in detail. In addition, placentas are readily available as medical waste after delivery, do not require any additional invasive procedure for collection, and do not pose ethical issues that plague other stem cell sources. More importantly, clinical research has indicated that the implantation of hAESCs or amniotic tissue in patients does not trigger an immune rejection, which might indicate their potential use as a solution to the graft-rejection-issue common in ESCs and iPSCs. Of note, preclinical experiments on animals have proven that hAESCs would not form tumors upon transplantation into either immunocompetent or immunodeficient mice, guaranteeing their safety for use in further clinical applications. Furthermore, as neonatal cells, hAESCs acquire little age- or environmental-associated DNA damage, indicating that they are genetically stable compared with human ESCs [183]. Since 150–200 million hAESCs can be obtained from a term placenta [184], there is no need to increase cell quantity by in vitro culture for single clinical application. On the other hand, with the fast development of the serum-free culture system, isolation and culturing methods for hAESCs have become normalized and convenient, making large-scale clinical use possible. Taken together, these properties make hAESCs attractive for cellular therapy.

However, some issues still need to be addressed. For one, a more exact mechanism of the immunomodulation process needs to be confirmed when hAESCs are transplanted in vivo. Although several cytokines and surface proteins have been proved to be expressed by hAESCs, further investigation is needed to determine how they work and the exact pathway. In addition, for those cells differentiated from hAESCs, more function tests need to be carried out to illuminate whether the cells are applicable for cell replacement, since the majority of studies have focused on the level of related marker detection rather than the cell function in vivo. For further clinical application, more research should be directed towards identifying the most suitable isolation markers for hAESC characterization to develop uniform standards. As described in recent study, the gestational age, region of cell isolation on placenta, isolation protocols, cross-contamination, passage number, epithelial-to-mesenchymal transition (EMT), and measuring methods are potential differences in the characterization of hAESCs and hAMSCs [185]. Alongside the rapid development of biomaterials aiming to support and protect cells being used for therapy, the combination of hAESCs and biomaterials is a promising tissue engineering method with special requirements.

In summary, as a promising cell source, hAESCs could potentially be used in clinical cell therapy. Although some details still need to be demonstrated, the unique advantages of hAESCs make them worthy of more attention and in-depth studies.

## Figures and Tables

**Table 1:** Isolation of amniotic epithelial stem cells (AESCs) from different species.

Species	Stage	Enzyme	Digestion Time	Cellular Yield	Reference
Rat	Embryonic day 15 (E15)	0.125% Trypsin	15 min	N.M.	[30]
Ovine	90 days of pregnancy	Trypsin	20 min	2–10 × 10^6^	[31]
Horse	N.M.	Collagenase type I + 0.25% Trypsin	3 h + 2 min	10 × 10^6^	[32]
Chicken	6-day-old chicken embryos	0.05% Trypsin	1 min + 5 min	N.M.	[33]
Feline	40–45 days of pregnancy	0.05% Trypsin	40 min	N.M.	[34]
Sheep	N.M.	0.25% Trypsin	30 min	N.M.	[35]

N.M., not mentioned.

**Table 2:** Differentiation potential of hAESCs (modified from reference [81]).

Lineage	Cell Type	Species	First Author	Procedure	Reference
Ectoderm	Neural progenitor cell	Human	Sakuragawa	N.M. (constitutive expression of some neural cell markers)	[43]
	Dopamine-producing cell	Human	Kakishita	100 μM L-tyrosine + 100 μM BH4 + 100μM MPT for 24 h, then transplanted into rat model of PD	[62]
	Neural cell	Human	Miki	5 × 10^−5^ M all-trans retinoic acid + 10 ng/mL FGF-4	[16]
	Oligodendrocyte	Human	Ishii	N.M. (constitutive expression of some oligodendrocyte cell markers)	[44]
	Neural cell	Human	Niknejad	1 μM ascorbic acid + 10 ng/mL bFGF + 100 ng/mL noggin for 21 days	[45]
	Neuronal	Rat	Okawa	N.M. (constitutive expression of some neural cell markers)	[46]
	Dopaminergic neuron	Human	Niknejad	1% insulin/transferrin/selenium (ITS), 2 mM ascorbic acid, 1% FCS (Sigma-Aldrich), 10 ng/mL bFGF (Sigma-Aldrich), and 100 ng/mL noggin for 7 days, then noggin is removed and cells are treated with 25 ng/mL FGF8 and 100 ng/mL Shh for maturation	[82]
	Progenitor of cortical neuron	Human	Lydia García-Castro	insulin/transferrin/selenium (ITS) supplemented with bFGF, EGF, SB431542, and noggin for 8 days, followed by serum-free N2 medium for 6 days	[83]
Mesoderm	Adipogenic	Human	Ilancheran	200 μM indomethacin + 0.5 mM isobutyl-methylxanthine + 1 μM dexamethasone + 10 μM insulin	[15]
	Chondrogenic	Human	Ilancheran	0.01 μM 1,25-dihydroxyvitamin D3 + 50 μM ascorbic acid + 10 mM b-glycerophosphate	[15]
	Cardiomyogenic	Human	Miki	1 mM ascorbic acid 2-phosphate for 14 days	[16]
	Cardiomyogenic	Rat	Fujimoto	N.M. (constitutive expression of some cardiac and vascular genes, in situ differentiation into vascular endothelial and smooth muscle cells after implantation)	[65]
	Osteoblast	Human	Si	DMEM supplemented with 10% FBS, ascorbic acid, β-glycerophosphate, and dexamethasone.	[84]
	Osteoblast	Human	Luan	Mechanical stretch stimulation and RNA interference	[85]
Endoderm	Hepatic	Human	Miki	10−7 M dexamethasone + 0.1 μM insulin, then 1 mM phenobarbital for 3 days	[16,73]
	Hepatic	Human	Sakuragawa	N.M. (constitutive expression of some genetic markers of liver lineage)	[86]
	Hepatic	Human	Takashima	N.M. (constitutive expression of a part of hepatocyte-related genes)	[87]
	Hepatic	Human	Manuelpillai	N.M. (transplant primary hAECs into mice and repair hepatic fibrosis)	[75]
	Hepatic	Human	Lin	Activin A for 5 days, followed by 5 days with bone morphogenetic protein 4 and fibroblast growth factor-2, then 5 days with hepatocyte growth factor, finally 3 days with oncostatin M and sodium taurocholate hydrate	[76]
	Hepatic	Human	Liu	10 ng/mL EGF and 10 ng/mL bFGF for 2 days, followed by 10 ng/mL EGF, 10 ng/mL bFGF, 20 ng/mL HGF, 1 μM dexamethasone, and 1% ITS Premix for 5 days; finally, 10 ng/mL EGF, 20 ng/mL oncostatin M, 20 ng/mL HGF, 1 μM dexamethasone, and 1% ITS Premix for 7 days	[74]
	Hepatic	Human	Furuya	use a 3D multicellular co-culture system	[88]
	Hepatic Sinusoidal Endothelial Cells	Human and rat	Serra	N.M. (differentiate in vivo)	[89]
	Insulin-producing cell	Human	Miki	10 mM nicotinamide for 14 days	[16]
	Insulin-producing cell	Human	Wei	N2 supplement + 10 mM nicotinamide for 2–4 weeks	[66]
	Insulin-producing cell	Human	Hou	N2 supplement + 10 mM nicotinamide for 2–4 weeks	[67]
	Insulin-producing cell	Human	Szukiewicz	10 mM nicotinamide	[68]
	Insulin-producing cell	Human	Okere	100 ng/mL activin A for 4 days in SFM, followed by 100 ng/mL activin A and 10 mM nicotinamide for 4 days, then 10 mM nicotinamide and 2 μM RA for 10 days	[70]
	Insulin-producing cell	Human	Luo	Stage 1: B27 supplement, activin A, and CHIR99021 for 1 day, followed by B27 with activin A for 2 days. Stage 2: B27 with dorsomorphin, retinoic acid, and SB431542 for 7 days. Stage 3: B27 contains forskolin, dexamethasone, Alk5 inhibitor II, and nicotinamide for 10 days.	[90]
	β islet-like cell	Human	Zou	serum-free DMEM containing N2 supplement and nicotinamide for 2 weeks	[91]
	Pancreatic progenitors	Human	Balaji	ectopic expression of PDX1	[92]
	Pneumocyte	Human	Moodley	N.M. (transplant primary hAECs into mice and differentiate in situ)	[41]

N.M., not mentioned.

**Table 3:** Clinical trials utilizing amnion membrane and hAESCs as biological intervention.

Drug	Category	Registration Number	Disease	Phases	Age	Country
Amnion Membrane	Ophthalmology	NCT02168790	Ocular Surface Disorders	Early 1	18–80 years	Germany
		NCT01341223	Ocular Surface Reconstruction	N/A	18–50 years	China
	Skin	NCT03754218	Burn	Early 1	18–65 years	United States
		ACTRN12619001050145	Burn	N/A	20–50 years	Pakistan
		ACTRN12618001632280	Skin Regeneration	N/A	20–45 years	Pakistan
		ACTRN12618001631291	Burn	N/A	10–40 years	Pakistan
Human Amniotic Epithelial Stem Cells (hAESCs)	Ophthalmology	NCT00344708	Corneal Epithelial Dystrophy	N/A	18–88 years	United States
	Neurology	NCT02961712	HTLV-1 Associated Myelopathy	1	18–75 years	China
		NCT03107975	Spastic Cerebral Palsy	1	1–5 years	China
		NCT04414813	Parkinson’s Disease	Early 1	30–70 years	China
		ACTRN12618000076279	Ischemic Stroke	1	18–85 years	Australia
	Gynecology	NCT02912104	Primary Ovarian Insufficiency, Premature Ovarian Failure	1	20–39 years	China
		NCT03223454	Asherman’s Syndrome	1	20–40 years	China
		NCT03207412	Premature Ovarian Failure	N/A	18–40 years	China
		NCT03381807	Intrauterine Adhesion	Early 1	20–45 years	China
	Pneumology	NCT02959333	Bronchial Fistula	1	18–75 years	China
		ACTRN12614000174684	Bronchopulmonary Dysplasia	1	36 weeks	Australia
		ACTRN12618000920291	Bronchopulmonary Dysplasia, Extremely Preterm Birth	1	14–18 days	Australia
		ACTRN12620000676910 p	COVID-19-Related Respiratory Failure	1/2	18–85 years	Australia
	Orthopedics	NCT03031509	Nonunion Fracture	1	18–80 years	China
	Others	NCT03764228	Acute Graft-Versus-Host Disease	N/A	18–70 years	China
		ACTRN12616000437460	Cirrhosis, Liver Fibrosis	1	18–70 years	Australia
		ACTRN12618001883202	Crohn’s Disease, Perianal Fistulas	N/A	18–80 years	Australia

N/A, not applicable.

## Abbreviations

AESCs	Amniotic epithelial stem cells
hAESCs	human amniotic epithelial stem cells
ESCs	Embryonic stem cells
AM	Amnion membrane
iPSCs	induced pluripotent stem cells
hAMSCs	human amniotic mesenchymal stromal cells
hUMSCs	human umbilical cord mesenchymal stromal cells
EGF	Epidermal growth factor
RA	Retinoic acid
HLA	Human leukocyte antigen
LSD	Lysosomal storage disease
MSCs	Mesenchymal stem cells
HMGB1	High-mobility group box 1
cffTF	cell-free fetal telomere fragments
OS	Oxidative stress
EMT	Epithelial to mesenchymal transition

## References

[1] Trounson A., McDonald C. (2015). Stem Cell Therapies in Clinical Trials: Progress and Challenges. Cell Stem Cell.

[2] Evans M.J., Kaufman M.H. (1981). Establishment in culture of pluripotential cells from mouse embryos. Nat. Cell Biol..

[3] Thomson J.A., Itskovitz-Eldor J., Shapiro S.S., Waknitz M.A., Swiergiel J.J., Marshall V.S., Jones J.M. (1998). Embryonic Stem Cell Lines Derived from Human Blastocysts. Science.

[4] Lumelsky N., Blondel O., Laeng P., Velasco I., Ravin R., McKay R. (2001). Differentiation of Embryonic Stem Cells to Insulin-Secreting Structures Similar to Pancreatic Islets. Science.

[5] Assady S., Maor G., Amit M., Itskovitz-Eldor J., Skorecki K.L., Tzukerman M. (2001). Insulin Production by Human Embryonic Stem Cells. Diabetes.

[6] Odorico J.S., Kaufman D.S., Thomson J.A. (2001). Multilineage Differentiation from Human Embryonic Stem Cell Lines. Stem Cells.

[7] Murry C.E., Keller G. (2008). Differentiation of Embryonic Stem Cells to Clinically Relevant Populations: Lessons from Embryonic Development. Cell.

[8] Klimanskaya I., Rosenthal N., Lanza R. (2008). Derive and conquer: Sourcing and differentiating stem cells for therapeutic applications. Nat. Rev. Drug Discov..

[9] Roy N.S., Cleren C., Singh S.K., Yang L., Beal M.F., Goldman S.A. (2006). Functional engraftment of human ES cell–derived dopaminergic neurons enriched by coculture with telomerase-immortalized midbrain astrocytes. Nat. Med..

[10] Zhao T., Zhang Z.-N., Rong Z., Xu Y. (2011). Immunogenicity of induced pluripotent stem cells. Nat. Cell Biol..

[11] Zhao T., Zhang Z.-N., Westenskow P.D., Todorova D., Hu Z., Lin T., Rong Z., Kim J., He J., Wang M. (2015). Humanized Mice Reveal Differential Immunogenicity of Cells Derived from Autologous Induced Pluripotent Stem Cells. Cell Stem Cell.

[12] Toda A., Okabe M., Yoshida T., Nikaido T. (2007). The Potential of Amniotic Membrane/Amnion-Derived Cells for Regeneration of Various Tissues. J. Pharmacol. Sci..

[13] De Coppi P., Atala A. (2019). Stem Cells from the Amnion. Principles of Regenerative Medicine.

[14] Antoniadou E., David A.L. (2016). Placental stem cells. Best Pr. Res. Clin. Obstet. Gynaecol..

[15] Ilancheran S., Michalska A., Peh G., Wallace E.M., Pera M., Manuelpillai U. (2007). Stem Cells Derived from Human Fetal Membranes Display Multilineage Differentiation Potential. Biol. Reprod..

[16] Miki T., Lehmann T., Cai H., Stolz D.B., Strom S.C. (2005). Stem Cell Characteristics of Amniotic Epithelial Cells. Stem Cells.

[17] Benirschke K., Kaufmann P., Baergen R.N. (2006). Pathology of the Human Placenta.

[18] Bryant-Greenwood G.D., Rees M.C.P., Turnbull A.C. (1987). Immunohistochemical localization of relaxin, prolactin and prostaglandin synthase in human amnion, chorion and decidua. J. Endocrinol..

[19] Okazaki T., Casey M.L., Okita J.R., Macdonald P.C., Johnston J.M. (1981). Initiation of human parturition. XII. Biosynthesis and metabolism of prostaglandins in human fetal membranes and uterine decidua. Am. J. Obstet. Gynecol..

[20] Lobo S.E., Leonel L.C.P., Miranda C.M., Coelho T.M., Ferreira G.A., Mess A., Abrão M.S., Miglino M.A. (2016). The Placenta as an Organ and a Source of Stem Cells and Extracellular Matrix: A Review. Cells Tissues Organs.

[21] Bryant-Greenwood G. (1998). The extracellular matrix of the human fetal membranes: Structure and function. Placenta.

[22] Bourne G. (1962). The F tal Membranes: A Review of the Anatomy of Normal Amnion and Chorion and Some Aspects of Their Function. Postgrad. Med. J..

[23] Calvin S.E., Oyen M.L. (2007). Microstructure and Mechanics of the Chorioamnion Membrane with an Emphasis on Fracture Properties. Ann. N. Y. Acad. Sci..

[24] Bourne G.L. (1966). The anatomy of the human amnion and chorion. Proc. R. Soc. Med..

[25] Cross J.C. (1998). Formation of the placenta and extraembryonic membranes. Ann. N. Y. Acad. Sci..

[26] Miki T., Strom S.C. (2006). Amnion-derived pluripotent/multipotent stem cells. Stem Cell Rev. Rep..

[27] Akle C., Welsh K., Adinolfi M., Leibowitz S., McColl I. (1981). Immunogenicity of Human Amniotic Epithelial Cells After Transplantation into Volunteers. Lancet.

[28] Gramignoli R., Srinivasan R.C., Kannisto K., Strom S.C. (2016). Isolation of Human Amnion Epithelial Cells According to Current Good Manufacturing Procedures. Curr. Protoc. Stem Cell Biol..

[29] Yang P.-J., Yuan W.-X., Liu J., Li J.-Y., Tan B., Qiu C., Zhu X.-L., Qiu C., Lai D., Guo L.-H. (2018). Biological characterization of human amniotic epithelial cells in a serum-free system and their safety evaluation. Acta Pharmacol. Sin..

[30] Shinya M., Komuro H., Saihara R., Urita Y., Kaneko M., Liu Y. (2010). Neural Differentiation Potential of Rat Amniotic Epithelial Cells. Fetal Pediatr. Pathol..

[31] Muttini A., Valbonetti L., Abate M., Colosimo A., Curini V., Mauro A., Berardinelli P., Russo V., Cocciolone D., Marchisio M. (2013). Ovine amniotic epithelial cells: In vitro characterization and transplantation into equine superficial digital flexor tendon spontaneous defects. Res. Veter-Sci..

[32] Lange-Consiglio A., Corradetti B., Bizzaro D., Magatti M., Ressel L., Tassan S., Parolini O., Cremonesi F. (2011). Characterization and potential applications of progenitor-like cells isolated from horse amniotic membrane. J. Tissue Eng. Regen. Med..

[33] Gao Y., Pu Y., Wang D., Zhang W., Guan W., Ma Y. (2012). Isolation and biological characterization of chicken amnion epithelial cells. Eur. J. Histochem..

[34] Rutigliano L., Corradetti B., Valentini L., Bizzaro D., Meucci A., Cremonesi F., Lange-Consiglio A. (2013). Molecular characterization and in vitro differentiation of feline progenitor-like amniotic epithelial cells. Stem Cell Res. Ther..

[35] Wu X., Gao F., Wu Y., Sun R., Guan W.-J., Tian X. (2019). Isolation and biological characteristics of sheep amniotic epithelial cells. Cytotechnology.

[36] Gaggi G., Di Credico A., Izzicupo P., Antonucci I., Crescioli C., Di Giacomo V., Di Ruscio A., Amabile G., Alviano F., Di Baldassarre A. (2020). Epigenetic Features of Human Perinatal Stem Cells Redefine Their Stemness Potential. Cells.

[37] Bryzek A., Czekaj P., Plewka D., Tomsia M., Komarska H., Lesiak M., Sieroń A.L., Sikora J., Kopaczka K. (2013). Expression and co-expression of surface markers of pluripotency on human amniotic cells cultured in different growth media. Ginekol. Polska.

[38] Miki T., Mitamura K., Ross M.A., Stolz N.B., Strom S.C. (2007). Identification of stem cell marker-positive cells by immunofluorescence in term human amnion. J. Reprod. Immunol..

[39] Han Y.M., Romero R., Kim J.-S., Tarca A.L., Kim S.K., Draghici S., Kusanovic J.P., Gotsch F., Mittal P., Hassan S.S. (2008). Region-Specific Gene Expression Profiling: Novel Evidence for Biological Heterogeneity of the Human Amnion. Biol. Reprod..

[40] Centurione L., Passaretta F., Centurione M.A., De Munari S., Vertua E., Silini A., Liberati M., Parolini O., Di Pietro R. (2018). Mapping of the Human Placenta. Cell Transplant..

[41] Moodley Y.P., Ilancheran S., Samuel C., Vaghjiani V., Atienza D., Williams E.D., Jenkin G., Wallace E., Trounson A., Manuelpillai U. (2010). Human Amnion Epithelial Cell Transplantation Abrogates Lung Fibrosis and Augments Repair. Am. J. Respir. Crit. Care Med..

[42] Tamagawa T., Ishiwata I., Saito S. (2008). Establishment and Characterization of a Pluripotent Stem Cell line Derived from Human Amniotic Membranes and Initiation of Germ Layers in vitro. Hum. Cell.

[43] Sakuragawa N., Thangavel R., Mizuguchi M., Hirasawa M., Kamo I. (1996). Expression of markers for both neuronal and glial cells in human amniotic epithelial cells. Neurosci. Lett..

[44] Ishii T., Ohsugi K., Nakamura S., Sato K., Hashimoto M., Mikoshiba K., Sakuragawa N. (1999). Gene expression of oligodendrocyte markers in human amniotic epithelial cells using neural cell-type-specific expression system. Neurosci. Lett..

[45] Niknejad H., Peirovi H., Ahmadiani A., Ghanavi J., Jorjani M. (2010). Differentiation factors that influence neuronal markers expression in vitro from human amniotic epithelial cells. Eur. Cells Mater..

[46] Okawa H., Okuda O., Arai H., Sakuragawa N., Sato K. (2001). Amniotic epithelial cells transform into neuron-like cells in the ischemic brain. Neuroreport.

[47] Marcus A.J., Coyne T.M., Black I.B., Woodbury D. (2008). Fate of amnion-derived stem cells transplanted to the fetal rat brain: Migration, survival and differentiation. J. Cell. Mol. Med..

[48] Dong W., Chen H., Yang X., Guo L., Hui G. (2011). Intraventricular transplantation of human amniotic epithelial cells on treatment of intracerebral hemorrhage model in rat. Chin. J. Neurosurg. Dis. Res..

[49] Xie H., Liu T., Guo L. (2007). Transplantation of human amniotic membrane epithelial cells and gene therapy in Parkinson’s disease rats. Chin. J. Cell Biol..

[50] Wu Z., Lu Y., Hui G., Wu X., Ji X., Huang Q., Guo L. (2006). Experimental study of human amniotic membrane cell transplantation for treatment of spinal cord injury in rats. Chin. J. Neurosurg..

[51] Lu Y., Hui G., Wu Z., Guo L., Ji X. (2006). Experimental study of transplantation of human amniotic membrane cells on traumatic brain injury in rats. Chin. J. Neurosurg..

[52] Sakuragawa N., Misawa H., Ohsugi K., Kakishita K., Ishii T., Thangavel R., Tohyama J., Elwan M., Yokoyama Y., Okuda O. (1997). Evidence for active acetylcholine metabolism in human amniotic epithelial cells: Applicable to intracerebral allografting for neurologic disease. Neurosci. Lett..

[53] Elwan M., Thangavel R., Ono F., Sakuragawa N. (1998). Synthesis and release of catecholamines by cultured monkey amniotic epithelial cells. J. Neurosci. Res..

[54] Elwan M.A. (1998). Synthesis of dopamine from L-3,4-dihydroxyphenylalanine by human amniotic epithelial cells. Eur. J. Pharmacol..

[55] Uchida S., Inanaga Y., Kobayashi M., Hurukawa S., Araie M., Sakuragawa N. (2000). Neurotrophic function of conditioned medium from human amniotic epithelial cells. J. Neurosci. Res..

[56] Koyano S., Fukui A., Uchida S., Yamada K., Asashima M., Sakuragawa N. (2002). Synthesis and release of activin and noggin by cultured human amniotic epithelial cells. Dev. Growth Differ..

[57] Meng X., Chen D., Dong Z., Liu J. (2007). Enhanced neural differentiation of neural stem cells and neurite growth by amniotic epithelial cell co-culture. Cell Biol. Int..

[58] Uchida S., Suzuki Y., Araie M., Kashiwagi K., Otori Y., Sakuragawa N. (2003). Factors secreted by human amniotic epithelial cells promote the survival of rat retinal ganglion cells. Neurosci. Lett..

[59] Meng X., Li C., Dong Z., Liu J., Li W., Liu Y., Xue H., Chen D. (2008). Co-transplantation of bFGF-expressing amniotic epithelial cells and neural stem cells promotes functional recovery in spinal cord-injured rats. Cell Biol. Int..

[60] Sankar V., Muthusamy R. (2003). Role of human amniotic epithelial cell transplantation in spinal cord injury repair research. Neuroscience.

[61] Arenas E. (2010). Towards stem cell replacement therapies for Parkinson’s disease. Biochem. Biophys. Res. Commun..

[62] Kakishita K., Elwan M.A., Nakao N., Itakura T., Sakuragawa N. (2000). Human Amniotic Epithelial Cells Produce Dopamine and Survive after Implantation into the Striatum of a Rat Model of Parkinson’s Disease: A Potential Source of Donor for Transplantation Therapy. Exp. Neurol..

[63] Kakishita K., Nakao N., Sakuragawa N., Itakura T. (2003). Implantation of human amniotic epithelial cells prevents the degeneration of nigral dopamine neurons in rats with 6-hydroxydopamine lesions. Brain Res..

[64] Yang X., Song L., Wu N., Liu Z., Xue S., Hui G. (2010). An experimental study on intracerebroventricular transplantation of human amniotic epithelial cells in a rat model of Parkinson’s disease. Neurol. Res..

[65] Fujimoto K.L., Miki T., Liu L.J., Hashizume R., Strom S.C., Wagner W.R., Keller B.B., Tobita K. (2009). Naive Rat Amnion-Derived Cell Transplantation Improved Left Ventricular Function and Reduced Myocardial Scar of Postinfarcted Heart. Cell Transplant..

[66] Szukiewicz D., Pyzlak M., Stangret A., Rongies W., Maslinska D. (2009). Decrease in expression of histamine H2 receptors by human amniotic epithelial cells during differentiation into pancreatic beta-like cells. Inflamm. Res..

[67] Hou Y., Huang Q., Liu T., Guo L. (2008). Human amnion epithelial cells can be induced to differentiate into functional insulin-producing cells. Acta Biochim. Biophys. Sin..

[68] Wei J.P., Zhang T.S., Kawa S., Aizawa T., Ota M., Akaike T., Kato K., Konishi I., Nikaido T. (2003). Human amnion-isolated cells normalize blood glucose in streptozotocin-induced diabetic mice. Cell Transplant..

[69] Skvorak K.J., Dorko K., Marongiu F., Tahan V., Hansel M.C., Gramignoli R., Gibson K.M., Strom S.C. (2013). Placental stem cell correction of murine intermediate maple syrup urine disease. Hepatology.

[70] Okere B., Alviano F., Costa R., Quaglino D., Ricci F., Dominici M., Paolucci P., Bonsi L., Iughetti L. (2015). In vitro differentiation of human amniotic epithelial cells into insulin-producing 3D spheroids. Int. J. Immunopathol. Pharmacol..

[71] Petersen B.E., Bowen W.C., Patrene K.D., Mars W.M., Sullivan A.K., Murase N., Boggs S.S., Greenberger J.S., Goff J.P. (1999). Bone Marrow as a Potential Source of Hepatic Oval Cells. Science.

[72] Chinzei R., Tanaka Y., Shimizu-Saito K., Hara Y., Kakinuma S., Watanabe M., Teramoto K., Arii S., Takase K., Sato C. (2002). Embryoid-body cells derived from a mouse embryonic stem cell line show differentiation into functional hepatocytes. Hepatology.

[73] Miki T., Marongiu F., Ellis E.C., Dorko K., Mitamura K., Ranade A., Gramignoli R., Davila J., Strom S.C. (2009). Production of Hepatocyte-Like Cells from Human Amnion. Protein-Ligand Inter..

[74] Xin H.-B., Liu Q.-Y., Li J.-Y., Wei L., Ren K.-K., Zhang X.-C., Ding T., Xiao L., Zhang W.-J., Wu H.-Y. (2018). Therapeutic efficiency of human amniotic epithelial stem cell-derived functional hepatocyte-like cells in mice with acute hepatic failure. Stem Cell Res. Ther..

[75] Lin J.S., Zhou L., Sagayaraj A., Jumat N.H.B., Choolani M., Chan J.K.Y., Biswas A., Wong P.C., Lim S.G., Dan Y.Y. (2015). Hepatic differentiation of human amniotic epithelial cells andin vivotherapeutic effect on animal model of cirrhosis. J. Gastroenterol. Hepatol..

[76] Manuelpillai U., Tchongue J., Lourensz D., Vaghjiani V., Samuel C.S., Liu A., Williams E.D., Sievert W. (2010). Transplantation of Human Amnion Epithelial Cells Reduces Hepatic Fibrosis in Immunocompetent CCl4-Treated Mice. Cell Transplant..

[77] Marongiu F., Gramignoli R., Dorko K., Miki T., Ranade A.R., Serra M.P., Doratiotto S., Sini M., Sharma S., Mitamura K. (2011). Hepatic differentiation of amniotic epithelial cells. Hepatology.

[78] Moritoki Y., Ueno Y., Kanno N., Yamagiwa Y., Fukushima K., Gershwin M.E., Shimosegawa T. (2007). Amniotic epithelial cell-derived cholangiocytes in experimental cholestatic ductal hyperplasia. Hepatol. Res..

[79] Zhou K., Koike C., Yoshida T., Okabe M., Fathy M., Kyo S., Kiyono T., Saito S., Nikaido T. (2013). Establishment and Characterization of Immortalized Human Amniotic Epithelial Cells. Cell. Reprogram..

[80] Han L.G., Zhao Q.-L., Yoshida T., Okabe M., Soko C., Rehman M.U., Kondo T., Nikaido T. (2019). Differential response of immortalized human amnion mesenchymal and epithelial cells against oxidative stress. Free. Radic. Biol. Med..

[81] Miki T. (2011). Amnion-derived stem cells: In quest of clinical applications. Stem Cell Res. Ther..

[82] Niknejad H., Deihim T., Ahmadiani A., Jorjani M., Peirovi H. (2012). Permanent expression of midbrain dopaminergic neurons traits in differentiated amniotic epithelial cells. Neurosci. Lett..

[83] García-Castro I.L., García-López G., Ávila-González D., Flores-Herrera H., Molina-Hernández A., Portillo W., Ramón-Gallegos E., Díaz N.F. (2015). Markers of Pluripotency in Human Amniotic Epithelial Cells and Their Differentiation to Progenitor of Cortical Neurons. PLoS ONE.

[84] Si J., Zhang J., Dai J., Yu D., Yu H., Shi J., Wang X., Shen S.G.F., Guo L. (2014). Osteogenic Differentiation of Human Amniotic Epithelial Cells and Its Application in Alveolar Defect Restoration. Stem Cells Transl. Med..

[85] Luan F., Ma K., Mao J., Yang F., Zhang M., Luan H. (2018). Differentiation of human amniotic epithelial cells into osteoblasts is induced by mechanical stretch via the Wnt/β-catenin signalling pathway. Mol. Med. Rep..

[86] Sakuragawa N., Enosawa S., Ishii T., Thangavel R., Tashiro T., Okuyama T., Suzuki S. (2000). Human amniotic epithelial cells are promising transgene carriers for allogeneic cell transplantation into liver. J. Hum. Genet..

[87] Takashima S., Ise H., Zhao P., Akaike T., Nikaido T. (2004). Human amniotic epithelial cells possess hepatocyte-like characteristics and functions. Cell Struct. Funct..

[88] Furuya K., Zheng Y.-W., Sako D., Iwasaki K., Zheng D.-X., Ge J.-Y., Liu L.-P., Furuta T., Akimoto K., Yagi H. (2019). Enhanced hepatic differentiation in the subpopulation of human amniotic stem cells under 3D multicellular microenvironment. World J. Stem Cells.

[89] Serra M., Marongiu M., Contini A., Miki T., Cadoni E., Laconi E., Marongiu F. (2018). Evidence of Amniotic Epithelial Cell Differentiation toward Hepatic Sinusoidal Endothelial Cells. Cell Transplant..

[90] Luo Y., Cheng Y.-W., Yu C.-Y., Liu R.-M., Zhao Y.-J., Chen D.-X., Zhong J.-J., Xiao J.-H. (2019). Effects of hyaluronic acid on differentiation of human amniotic epithelial cells and cell-replacement therapy in type 1 diabetic mice. Exp. Cell Res..

[91] Zou G., Liu T., Guo L., Huang Y., Feng Y., Duan T. (2018). MicroRNA-32 silences WWP2 expression to maintain the pluripotency of human amniotic epithelial stem cells and β islet-like cell differentiation. Int. J. Mol. Med..

[92] Balaji S., Zhou Y., Ganguly A., Opara E.C., Soker S. (2016). The combined effect of PDX1, epidermal growth factor and poly-L-ornithine on human amnion epithelial cells’ differentiation. BMC Dev. Biol..

[93] Lynch M.E., Chiou A.E., Lee M.J., Marcott S.C., Polamraju P.V., Lee Y., Fischbach C. (2016). Three-Dimensional Mechanical Loading Modulates the Osteogenic Response of Mesenchymal Stem Cells to Tumor-Derived Soluble Signals. Tissue Eng. Part A.

[94] Niknejad H., Khayat-Khoei M., Peirovi H., Abolghasemi H. (2014). Human amniotic epithelial cells induce apoptosis of cancer cells: A new anti-tumor therapeutic strategy. Cytotherapy.

[95] Choi K.-C., Kang N.-H., Yi B.-R., Lim S.Y., Hwang K.-A., Baek Y.S. (2012). Human amniotic membrane-derived epithelial stem cells display anticancer activity in BALB/c female nude mice bearing disseminated breast cancer xenografts. Int. J. Oncol..

[96] Bu S., Zhang Q., Wang Q., Lai D. (2017). Human amniotic epithelial cells inhibit growth of epithelial ovarian cancer cells via TGF-β1-mediated cell cycle arrest. Int. J. Oncol..

[97] Medawar P.B. (1953). Some immunological and endocrinological problems raised by the evolution of viviparity. Symp. Soc. Exp. Biol..

[98] Lapaire O., Holzgreve W., Oosterwijk J.C., Brinkhaus R., Bianchi D.W. (2007). Georg Schmorl on Trophoblasts in the Maternal Circulation. Placenta.

[99] Lo Y.D., Chan K.C.A., Sun H., Chen E.Z., Jiang P., Lun F.M.F., Zheng Y.W., Leung T.Y., Lau T.K., Cantor C.R. (2010). Maternal Plasma DNA Sequencing Reveals the Genome-Wide Genetic and Mutational Profile of the Fetus. Sci. Transl. Med..

[100] Prabhudas M., Bonney E., Caron K., Dey S., Erlebacher A., Fazleabas A., Fisher S., Golos T., Matzuk M., McCune J.M. (2015). Immune mechanisms at the maternal-fetal interface: Perspectives and challenges. Nat. Immunol..

[101] Kubo M., Sonoda Y., Muramatsu R., Usui M. (2001). Immunogenicity of human amniotic membrane in experimental xenotransplantation. Investig. Ophthalmol. Vis. Sci..

[102] Yuge I., Takumi Y., Koyabu K., Hashimoto S., Takashima S., Fukuyama T., Nikaido T., Usami S.-I. (2004). Transplanted Human Amniotic Epithelial Cells Express Connexin 26 and Na-K-Adenosine Triphosphatase in the Inner EAR. Transplantation.

[103] Hori J., Wang M., Kamiya K., Takahashi H., Sakuragawa N. (2006). Immunological Characteristics of Amniotic Epithelium. Cornea.

[104] Adinolfi M., Akle C.A., McColl I., Fensom A.H., Tansley L., Connolly P., Hsi B.-L., Faulk W.P., Travers P., Bodmer W.F. (1982). Expression of HLA antigens, β2-microglobulin and enzymes by human amniotic epithelial cells. Nat. Cell Biol..

[105] Rylova Y.V., Milovanova N.V., Gordeeva M.N., Savilova A.M. (2015). Characteristics of Multipotent Mesenchymal Stromal Cells from Human Terminal Placenta. Bull. Exp. Biol. Med..

[106] Hammer A., Hutter H., Blaschitz A., Mahnert W., Hartmann M., Uchańska-Ziegler B., Ziegler A., Dohr G. (1997). Amnion Epithelial Cells, in Contrast to Trophoblast Cells, Express All Classical HLA Class I Molecules Together With HLA-G. Am. J. Reprod. Immunol..

[107] Parolini O., Alviano F., Bagnara G.P., Bilic G., Bühring H.-J., Evangelista M., Hennerbichler S., Liu B., Magatti M., Mao N. (2008). Concise Review: Isolation and Characterization of Cells from Human Term Placenta: Outcome of the First International Workshop on Placenta Derived Stem Cells. Stem Cells.

[108] Wolbank S., Peterbauer A., Fahrner M., Hennerbichler S., Van Griensven M., Stadler G., Redl H., Gabriel C. (2007). Dose-Dependent Immunomodulatory Effect of Human Stem Cells from Amniotic Membrane: A Comparison with Human Mesenchymal Stem Cells from Adipose Tissue. Tissue Eng..

[109] Pratama G., Vaghjiani V., Tee J.Y., Liu Y.H., Chan J., Tan C., Murthi P., Gargett C., Manuelpillai U. (2011). Changes in Culture Expanded Human Amniotic Epithelial Cells: Implications for Potential Therapeutic Applications. PLoS ONE.

[110] Strom S.C., Gramignoli R. (2016). Human amnion epithelial cells expressing HLA-G as novel cell-based treatment for liver disease. Hum. Immunol..

[111] Fainardi E., Castellazzi M., Stignani M., Morandi F., Sana G., Gonzalez R., Pistoia V., Baricordi O.R., Sokal E., Peña J. (2010). Emerging topics and new perspectives on HLA-G. Cell. Mol. Life Sci..

[112] Hunt J.S., Petroff M.G., McIntire R.H., Ober C. (2005). HLA-G and immune tolerance in pregnancy. FASEB J..

[113] Ellis S.A., Sargent I.L., Redman C.W., McMichael A.J. (1986). Evidence for a novel HLA antigen found on human extravillous trophoblast and a choriocarcinoma cell line. Immunology.

[114] Banas R.A., Trumpower C., Bentlejewski C., Marshall V., Sing G., Zeevi A. (2008). Immunogenicity and immunomodulatory effects of amnion-derived multipotent progenitor cells. Hum. Immunol..

[115] Lim R., Chan S., Tan J., Mockler J., Murphy S., Wallace E.M. (2013). Preterm human amnion epithelial cells have limited reparative potential. Placenta.

[116] Riteau B., Menier C., Khalil-Daher I., Sedlik C., Dausset J., Rouas-Freiss N., Carosella E.D. (1999). HLA-G inhibits the allogeneic proliferative response. J. Reprod. Immunol..

[117] Le Maux A., Noël G., Birebent B., Grosset J., Vu N., De Guibert S., Bernard M., Sémana G., Amiot L. (2008). Soluble human leucocyte antigen-G molecules in peripheral blood haematopoietic stem cell transplantation: A specific role to prevent acute graft-versus-host disease and a link with regulatory T cells. Clin. Exp. Immunol..

[118] Gonen-Gross T., Goldman-Wohl D., Huppertz B., Lankry D., Greenfield C., Natanson-Yaron S., Hamani Y., Gilad R., Yagel S., Mandelboim O. (2010). Inhibitory NK Receptor Recognition of HLA-G: Regulation by Contact Residues and by Cell Specific Expression at the Fetal-Maternal Interface. PLoS ONE.

[119] Krampera M. (2011). Mesenchymal stromal cell ‘licensing’: A multistep process. Leukemia.

[120] Gillaux C., Méhats C., Vaiman D., Cabrol D., Breuiller-Fouché M. (2011). Functional Screening of TLRs in Human Amniotic Epithelial Cells. J. Immunol..

[121] Goldenberg R.L., Culhane J.F., Iams J.D., Romero R. (2008). Epidemiology and causes of preterm birth. Lancet.

[122] Inge E., Talmi Y.P., Sigler L., Finkelstein Y., Zohar Y. (1991). Antibacterial properties of human amniotic membranes. Placenta.

[123] Galask R.P., Snyder I.S. (1970). Antimicrobial factors in amniotic fluid. Am. J. Obstet. Gynecol..

[124] Buhimschi I.A., Jabr M., Buhimschi C.S., Petkova A.P., Weiner C.P., Saed G.M. (2004). The novel antimicrobial peptide β3-defensin is produced by the amnion: A possible role of the fetal membranes in innate immunity of the amniotic cavity. Am. J. Obstet. Gynecol..

[125] Itoh N., Yonehara S., Ishii A., Yonehara M., Mizushima S.-I., Sameshima M., Hase A., Seto Y., Nagata S. (1991). The polypeptide encoded by the cDNA for human cell surface antigen Fas can mediate apoptosis. Cell.

[126] Cheng J., Zhou T., Liu C., Shapiro J.P., Brauer M.J., Kiefer M.C., Barr P.J., Mountz J.D., Bolshakov V., Siegelbaum S. (1994). Protection from Fas-mediated apoptosis by a soluble form of the Fas molecule. Science.

[127] Hsu C.-D., Hong S.-F., Harirah H., Bahado-Singh R., Lu L.-C. (2000). Amniotic fluid soluble fas levels in intra-amniotic infection. Obstet. Gynecol..

[128] Harirah H., Donia S.E., Parkash V., Jones D.C., Hsu C.-D. (2002). Localization of the Fas-Fas ligand system in human fetal membranes. J. Reprod. Med..

[129] Liu Y.H., Vaghjiani V., Tee J.Y., To K., Cui P., Oh D.Y., Manuelpillai U., Toh B.-H., Chan J. (2012). Amniotic Epithelial Cells from the Human Placenta Potently Suppress a Mouse Model of Multiple Sclerosis. PLoS ONE.

[130] Bailo M., Soncini M., Vertua E., Signoroni P.B., Sanzone S., Lombardi G., Arienti D., Calamani F., Zatti D., Paul P. (2004). Engraftment Potential of Human Amnion and Chorion Cells Derived from Term Placenta. Transplantation.

[131] Tan J., Chan S.T., Lo C.Y., Deane J.A., McDonald C.A., Bernard C.C., Wallace E.M., Lim R. (2015). Amnion cell-mediated immune modulation following bleomycin challenge: Controlling the regulatory T cell response. Stem Cell Res. Ther..

[132] Li J., Koike-Soko C., Sugimoto J., Yoshida T., Okabe M., Nikaido T. (2015). Human Amnion-Derived Stem Cells Have Immunosuppressive Properties on NK Cells and Monocytes. Cell Transplant..

[133] Alipour R., Motedayyen H., Sereshki N., Rafiee M., Alsahebfosul F., Pourazar A. (2020). Human Amniotic Epithelial Cells Affect the Functions of Neutrophils. Int. J. Stem Cells.

[134] Li J., Qiu C., Zhang Z., Yuan W., Ge Z., Tan B., Yang P., Liu J., Zhu X., Qiu C. (2018). Subretinal Transplantation of Human Amniotic Epithelial Cells in the Treatment of Autoimmune Uveitis in Rats. Cell Transplant..

[135] Tan B., Yuan W., Li J., Yang P., Ge Z., Liu J., Qiu C., Zhu X., Qiu C., Lai D. (2018). Therapeutic effect of human amniotic epithelial cells in murine models of Hashimoto’s thyroiditis and Systemic lupus erythematosus. Cytotherapy.

[136] Li H., Niederkorn J.Y., Neelam S., Mayhew E., Word R.A., McCulley J.P., Alizadeh H. (2005). Immunosuppressive Factors Secreted by Human Amniotic Epithelial Cells. Investig. Opthalmol. Vis. Sci..

[137] Hao Y., Ma D.H.-K., Hwang D.G., Kim W.-S., Zhang F. (2000). Identification of Antiangiogenic and Antiinflammatory Proteins in Human Amniotic Membrane. Cornea.

[138] Kamiya K., Wang M., Uchida S., Amano S., Oshika T., Sakuragawa N., Hori J. (2005). Topical application of culture supernatant from human amniotic epithelial cells suppresses inflammatory reactions in cornea. Exp. Eye Res..

[139] Rooney I.A., Morgan B.P. (1990). Protection of human amniotic epithelial cells (HAEC) from complement-mediated lysis: Expression on the cells of three complement inhibitory membrane proteins. Immunology.

[140] Nicholson-Weller A., Burge J., Fearon D.T., Weller P.F., Austen K.F. (1982). Isolation of a human erythrocyte membrane glycoprotein with decay-accelerating activity for C3 convertases of the complement system. J. Immunol..

[141] Zalman L.S., Wood L.M., Muller-Eberhard H.J. (1986). Isolation of a human erythrocyte membrane protein capable of inhibiting expression of homologous complement transmembrane channels. Proc. Natl. Acad. Sci. USA.

[142] Davies A., Simmons D.L., Hale G., Harrison R.A., Tighe H., Lachmann P., Waldmann H. (1989). CD59, an LY-6-like protein expressed in human lymphoid cells, regulates the action of the complement membrane attack complex on homologous cells. J. Exp. Med..

[143] Sheller-Miller S., Papaconstantinou J., Urrabaz-Garza R., Richardson L., Saade G., Salomon C., Menon R. (2016). Amnion-Epithelial-Cell-Derived Exosomes Demonstrate Physiologic State of Cell under Oxidative Stress. PLoS ONE.

[144] Sheller-Miller S., Urrabaz-Garza R., Saade G.R., Menon R. (2017). Damage-Associated molecular pattern markers HMGB1 and cell-Free fetal telomere fragments in oxidative-Stressed amnion epithelial cell-Derived exosomes. J. Reprod. Immunol..

[145] Hadley E.E., Sheller-Miller S., Saade G., Salomon C., Mesiano S., Taylor R.N., Taylor B.D., Menon R. (2018). Amnion epithelial cell-derived exosomes induce inflammatory changes in uterine cells. Am. J. Obstet. Gynecol..

[146] Zhao B., Zhang Y., Han S., Zhang W., Zhou Q., Guan H., Liu J., Shi J., Su L., Hu D. (2017). Exosomes derived from human amniotic epithelial cells accelerate wound healing and inhibit scar formation. J. Mol. Histol..

[147] Zhao B., Li X., Shi X., Shi X., Zhang W., Wu G., Wang X., Su L.-L., Hu D. (2018). Exosomal MicroRNAs Derived from Human Amniotic Epithelial Cells Accelerate Wound Healing by Promoting the Proliferation and Migration of Fibroblasts. Stem Cells Int..

[148] Wei P., Zhong C., Yang X., Shu F., Xiao S., Gong T., Luo P., Li L., Chen Z., Zheng Y. (2020). OUP accepted manuscript. Burn. Trauma.

[149] Tan J.L., Lau S.N., Leaw B., Nguyen H.P.T., Salamonsen L.A., Saad M.I., Chan S.T., Zhu D., Krause M., Kim C. (2018). Amnion Epithelial Cell-Derived Exosomes Restrict Lung Injury and Enhance Endogenous Lung Repair. Stem Cells Transl. Med..

[150] Alhomrani M., Correia J., Zavou M., Leaw B., Kuk N., Xu R., Saad M.I., Hodge A., Greening D.W., Lim R. (2017). The Human Amnion Epithelial Cell Secretome Decreases Hepatic Fibrosis in Mice with Chronic Liver Fibrosis. Front. Pharmacol..

[151] Vosdoganes P., Wallace E.M., Chan S.T., Acharya R., Moss T.J., Lim R. (2013). Human Amnion Epithelial Cells Repair Established Lung Injury. Cell Transplant..

[152] Murphy S.V., Shiyun S.C., Tan J., Chan S., Jenkin G., Wallace E.M., Lim R. (2012). Human Amnion Epithelial Cells do Not Abrogate Pulmonary Fibrosis in Mice with Impaired Macrophage Function. Cell Transplant..

[153] Tan J., Chan S.T., Wallace E.M., Lim R. (2014). Human Amnion Epithelial Cells Mediate Lung Repair by Directly Modulating Macrophage Recruitment and Polarization. Cell Transplant..

[154] Tan J.L., Tan Y.Z., Muljadi R., Chan S.T., Lau S.N., Mockler J.C., Wallace E.M., Lim R. (2016). Amnion Epithelial Cells Promote Lung Repair via Lipoxin A. Stem Cells Transl. Med..

[155] Kim K.Y., Suh Y.-H., Chang K.-A. (2020). Therapeutic Effects of Human Amniotic Epithelial Stem Cells in a Transgenic Mouse Model of Alzheimer’s Disease. Int. J. Mol. Sci..

[156] Zheng Y., Zheng S., Fan X., Li L., Xiao Y., Luo P., Liu Y., Wang L., Cui Z., He F. (2018). Amniotic Epithelial Cells Accelerate Diabetic Wound Healing by Modulating Inflammation and Promoting Neovascularization. Stem Cells Int..

[157] Manuelpillai U., Lourensz D., Vaghjiani V., Tchongue J., Lacey D., Tee J.-Y., Murthi P., Chan J., Hodge A., Sievert W. (2012). Human Amniotic Epithelial Cell Transplantation Induces Markers of Alternative Macrophage Activation and Reduces Established Hepatic Fibrosis. PLoS ONE.

[158] Cargnoni A., Farigu S., Piccinelli E.C., Signoroni P.B., Romele P., Vanosi G., Toschi I., Cesari V., Sant’Anna L.B., Magatti M. (2017). Effect of human amniotic epithelial cells on pro-fibrogenic resident hepatic cells in a rat model of liver fibrosis. J. Cell. Mol. Med..

[159] Andrewartha N., Yeoh G.C.T. (2019). Human Amnion Epithelial Cell Therapy for Chronic Liver Disease. Stem Cells Int..

[160] Bai X., Liu J., Yuan W., Liu Y., Li W., Cao S., Yu L., Wang L. (2020). Therapeutic Effect of Human Amniotic Epithelial Cells in Rat Models of Intrauterine Adhesions. Cell Transplant..

[161] Li B., Zhang Q., Sun J., Lai D. (2019). Human amniotic epithelial cells improve fertility in an intrauterine adhesion mouse model. Stem Cell Res. Ther..

[162] Ouyang M.X., You S., Zhang Y., Zhang C., Zhang G., Shao X., He F., Hu L. (2020). Transplantation of Human Amnion Epithelial Cells Improves Endometrial Regeneration in Rat Model of Intrauterine Adhesions. Stem Cells Dev..

[163] Canciello A., Russo V., Berardinelli P., Bernabò N., Muttini A., Mattioli M., Barboni B. (2017). Progesterone prevents epithelial-mesenchymal transition of ovine amniotic epithelial cells and enhances their immunomodulatory properties. Sci. Rep..

[164] Hou S., Ding C., Shen H., Qian C., Zou Q., Lu J., Huang B., Tan J., Li H. (2020). Vitamin C improves the therapeutic potential of human amniotic epithelial cells in premature ovarian insufficiency disease. Stem Cell Res. Ther..

[165] Davis S. (1910). Skin Transplantation: With a Review of 550 Cases at the Johns Hopkins Hospital. Johns Hopkins Med. J..

[166] Dhall K. (1984). Amnion graft for treatment of congenital absence of the vagina. BJOG Int. J. Obstet. Gynaecol..

[167] Troensegaard-Hansen E. (1950). Amniotic Grafts in Chronic Skin Ulceration. Lancet.

[168] Faulk W., Stevens P., Burgos H., Matthews R., Bennett J., Hsi B.-L. (1980). Human Amnion as an Adjunct in Wound Healing. Lancet.

[169] Stern M. (1913). The Grafting of Preserved Amniotic Membrane to Burned and Ulcerated Surfaces, Substituing Skin Grafts. J. Am. Med. Assoc..

[170] Gharib M., Ure B.M., Klose M. (1996). Use of amniotic grafts in the repair of gastroschisis. Pediatr. Surg. Int..

[171] Gomes J.Á.P., Romano A., Santos M.S., Dua H.S. (2005). Amniotic membrane use in ophthalmology. Curr. Opin. Ophthalmol..

[172] Dua H.S., Gomes J.A., King A.J., Maharajan V. (2004). The amniotic membrane in ophthalmology. Surv. Ophthalmol..

[173] Yeager A.M., Singer H.S., Buck J.R., Matalon R., Brennan S., O’Toole S.O., Moser H.W., Opitz J.M., Reynolds J.F. (1985). A therapeutic trial of amniotic epithelial cell implantation in patients with lysosomal storage diseases. Am. J. Med. Genet..

[174] Akle C., McColl I., Dean M., Adinolfi M., Brown S., Fensom A.H., Marsh J., Welsh K. (1985). Transplantation of amniotic epithelial membranes in patients with mucopolysaccharidoses. Exp. Clin. Immunogenet..

[175] Scaggiante B., Pineschi A., Sustersich M., Andolina M., Agosti E., Romeo D. (1987). Successful Therapy of Niemann-Pick Disease by Implantation of Human Amniotic Membrane. Transplantation.

[176] Bembi B., Comelli M., Scaggiante B., Pineschi A., Rapelli S., Gornati R., Montorfano G., Berra B., Agosti E., Romeo D. (1992). Treatment of sphingomyelinase deficiency by repeated implantations of amniotic epithelial cells. Am. J. Med. Genet..

[177] Sakuragawa N., Yoshikawa H., Sasaki M. (1992). Amniotic tissue transplantation: Clinical and biochemical evaluations for some lysosomal storage diseases. Brain Dev..

[178] Tylki-Szymańska A., Maciejko D., Kidawa M., Jabłońska-Budaj U., Czartoryska B. (1985). Amniotic tissue transplantation as a trial of treatment in some lysosomal storage diseases. J. Inherit. Metab. Dis..

[179] Scaggiante B., Pineschi A., Sustersich M., Andolina M., Agosti E., Romeo D., Marzari R. (1987). Graft of cryopreserved human amniotic epithelial cells in a subject with type B Niemann-Pick disease. Pediatr. Med. Chir..

[180] Parmar D.N., Alizadeh H., Awwad S.T., Li H., Neelam S., Bowman R.W., Cavanagh H.D., McCulley J.P. (2006). Ocular Surface Restoration Using Non-Surgical Transplantation of Tissue-Cultured Human Amniotic Epithelial Cells. Am. J. Ophthalmol..

[181] Lim R., Malhotra A., Tan J., Chan S.T., Lau S., Zhu D., Mockler J.C., Wallace E.M. (2018). First-In-Human Administration of Allogeneic Amnion Cells in Premature Infants With Bronchopulmonary Dysplasia: A Safety Study. Stem Cells Transl. Med..

[182] Baradaran-Rafii A., Asl N.S., Ebrahimi M., Jabbehdari S., Bamdad S., Roshandel D., Eslani M., Momeni M. (2018). The role of amniotic membrane extract eye drop (AMEED) in in vivo cultivation of limbal stem cells. Ocul. Surf..

[183] Easley C.A., Miki T., Castro C.A., Ozolek J.A., Minervini C.F., Ben-Yehudah A., Schatten G. (2012). Human Amniotic Epithelial Cells are Reprogrammed More Efficiently by Induced Pluripotency than Adult Fibroblasts. Cell. Reprogram..

[184] Miki T. (2018). Stem cell characteristics and the therapeutic potential of amniotic epithelial cells. Am. J. Reprod. Immunol..

[185] Ghamari S.-H., Abbasi-Kangevari M., Tayebi T., Bahrami S., Niknejad H. (2020). The Bottlenecks in Translating Placenta-Derived Amniotic Epithelial and Mesenchymal Stromal Cells Into the Clinic: Current Discrepancies in Marker Reports. Front. Bioeng. Biotechnol..

